# How Online Reviews and Services Affect Physician Outpatient Visits: Content Analysis of Evidence From Two Online Health Care Communities

**DOI:** 10.2196/16185

**Published:** 2019-12-02

**Authors:** Wei Lu, Hong Wu

**Affiliations:** 1 School of Medicine and Health Management Tongji Medical College Huazhong University of Science and Technology Wuhan China

**Keywords:** online health care communities, online reviews, online services, outpatient care, channel effect, patient choice

## Abstract

**Background:**

Online healthcare communities are changing the ways of physician-patient communication and how patients choose outpatient care physicians. Although a majority of empirical work has examined the role of online reviews in consumer decisions, less research has been done in health care, and endogeneity of online reviews has not been fully considered. Moreover, the important factor of physician online services has been neglected in patient decisions.

**Objective:**

In this paper, we addressed the endogeneity of online reviews and examined the impact of online reviews and services on outpatient visits based on theories of reviews and channel effects.

**Methods:**

We used a difference-in-difference approach to account for physician- and website-specific effects by collecting information from 474 physician homepages on two online health care communities.

**Results:**

We found that the number of reviews was more effective in influencing patient decisions compared with the overall review rating. An improvement in reviews leads to a relative increase in physician outpatient visits on that website. There are channel effects in health care: online services complement offline services (outpatient care appointments). Results further indicate that online services moderate the relationship between online reviews and physician outpatient visits.

**Conclusions:**

This study investigated the effect of reviews and channel effects in health care by conducting a difference-in-difference analysis on two online health care communities. Our findings provide basic research on online health care communities.

## Introduction

### Background

Patients often face uncertainty regarding the quality of physician services such as medical quality and bedside manner and often lack channels to access that information [1]. Information disclosure of medical quality is mainly based on the hospital/nursing home/organization level. However, patients are increasingly concerned about health care quality at the physician level. Information asymmetries between patients and physicians are extensive. Traditionally, patients relied on social networks to learn this information, such as peer recommendations. With the growing popularity of Web 2.0 technologies, online health care communities provide a useful channel for people to get physician information and have become an integral part of their daily lives. In 2013, the number of adults who used the internet to search online for health care information was 59% in the United States [2]. More than 80% of patients search for health information before going to the doctor in China [3].

Online health care communities provide review forums, in which patients can share their disease information and treatment experience with other members of the community. In the absence of other channels to acquire information on physician medical quality, online review forums provide a potential opportunity for patients. Compared with traditional channels (eg, acquaintance recommendations), however, there has not been enough research into whether patients trust and refer to this information received online from strangers. Much effort has been dedicated to researching the health care quality of organizations such as hospitals and nursing homes [4,5], but less has been done at the individual physician level. Moreover, although quite a few studies have investigated the relationship between reviews and performance and generally get consistent results that higher reviews correlate with improved performance in other fields [6-8], the endogeneity of online reviews that may cause bias has not been fully considered in previous studies.

Today’s organizations are continually adding new marketing channels through the internet to better serve their products and/or service receivers [9], and this phenomenon is also manifested in the health care industry. Online health care communities enable physicians to better help and serve patients by providing physicians with a variety of functions—for example, question and answer (written consultation) and telephone consultation services. With channel diversification, researchers try to find channel effects and channel choice [10,11]. Some researchers assert that the internet competes with traditional channels by decreasing transaction costs, such as search and monitoring [12,13]. For example, service receivers could find service providers in distant geographic markets who have lower prices, provide better service, offer higher quality products, or have products that better match their needs [12,13]. However, other researchers emphasize the importance of synergies between online and offline channels [14,15], demonstrating that the use of multiple channels tends to be more successful. Online channels have spillover effects, generating increased purchases in offline channels [16]. However, there are only a few studies that empirically explore the channel effect, especially in the health field [11].

Both online reviews and online services provide information sources for patients. Online reviews help patients get information about the treatment experience from others, and patients can use online services to get their own experience by communicating with physicians directly. When physicians choose to provide online services, this may decrease patient dependence on online reviews. With the development of online channels, efforts to examine whether there are moderating effects of online services on the relationship between reviews and offline service (ie, outpatient visits) becomes necessary. The specific research questions addressed in this paper are as follows:

- RQ1: How do online reviews impact physician outpatient visits?
- RQ2: How do physician services provided via online channels impact their outpatient visits?
- RQ3: How does the relationship between online reviews and outpatient visits change relative to physician online services?

**Figure 1:**
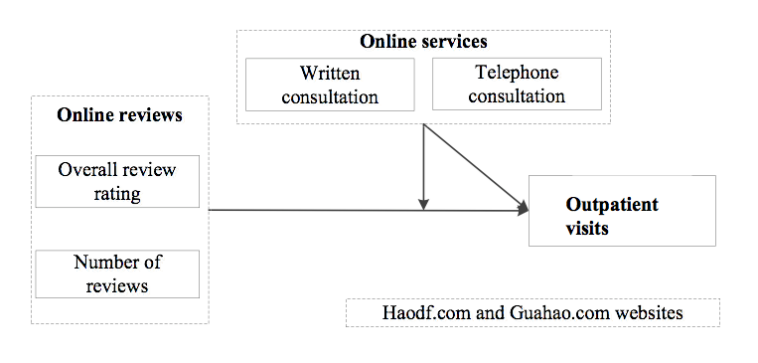
Conceptual model.

To answer these questions and solve endogeneity issues in the empirical estimation, we used a dataset of 474 physicians from two leading online health care communities, Haodf [17] and Guahao [18], to construct measures of each physician’s outpatient visits and used a difference-in-difference approach to account for physician- and website-specific effects. Both Haodf and Guahao allow patients to post reviews on their platforms. In addition to outpatient visits, physicians on Haodf can provide online services (written and telephone consultations) for patients. Guahao only provides outpatient appointments. We use the overall review rating and number of reviews to measure the quality of reviews, both of which have been used in prior studies and are considered to be useful [1,19]. A difference-in-difference approach similar to that used in Chevalier and Mayzlin [20] is used in our paper: we measured reviews and number of outpatient visits for each physician who works at both Haodf and Guahao over three time points, and we examined whether a change in overall review rating and number of reviews over time for a physician on one website relative to the other predicts a change in subsequent outpatient visits of that physician on one website relative to the other. By using this approach, we were able to control for possible effects of unobserved physician characteristics on both reviews and outpatient visits. Moreover, by focusing on the differences across websites over time, we controlled for the unobserved website fixed effects at the two websites that may have affected both reviews and outpatient visits, such as website design, different patient populations, and patient preference. Figure 1 shows the conceptual model of this study. The hypotheses, presented below, were established according to the relationships expressed in the model in Figure 1.

### Online Health Care Communities

With the growing popularity of Web 2.0 technologies such as blogs, tweets, podcasts, and wikis, many health care organizations and professionals are embracing social media. The use of social network software, with its ability to enrich the connection between patients and the rest of the medical industry, has been dubbed Health 2.0 [21], and the number of organizations adopting Health 2.0 is growing. Many online communities have been developed by patient organizations, providers, and nonprofit organizations in recent years, making it easier for patients to find health information [22,23]. Such online communities are virtual forums for patients to discuss their health concerns, share information about treatments and support, and communicate with physicians, an example of which is PatientsLikeMe [24].

Researchers have started to investigate the benefits of online health care communities for physicians [25,26] and patients [27]. Xiao et al [28] examined the factors that influence patients’ online health care information searches and found that perceived health status could affect patients’ online health care search frequency and diversity. Privacy concerns, trust, and information sensitivity are factors that have an impact on people’s decisions about whether to place their health information online [29].

As a result of the limitations of existing health services, online communities in China have emerged in recent years. China has the world’s largest population and thus represents a huge resource-consumption country. China’s large population generates a variety of unique needs relating to medical services, therefore exhibiting unique behaviors within online health care communities. Health intimately concerns everyone, and with the emergence of online health care communities, patients have more channels to get physician information and physicians have more choices in the ways of helping patients. Based on the existing literature, we have found few studies exploring the causal effects of online reviews and services on outpatient visits. Our study aims to fill these gaps.

### Endogeneity of Online Reviews

Several factors cause the endogeneity of online reviews. First, whether to post reviews is self-selected. Existing studies suggest that consumers are motivated to engage in posting reviews for different reasons (altruistic, product involvement, self-enhancement purposes, anxiety-reducing, vengeful, and advice-seeking, etc) [30,31]. This kind of volitional activity is likely subject to a variety of biases and social influences [32,33] that will cause estimation deviation if not considered. Second, product and service quality can be an underlying factor that drives both reviews and sales. Research has shown that consumers who are particularly satisfied or dissatisfied with the product or service quality will post feedback to let other knows [34]. Product and service quality is often hard to quantify or observe, especially in health care. Researchers face difficulties deciding whether high reviews or high product/service quality impacts high visits [35]. In this paper, we attempt to resolve the above endogeneity problem using a sophisticated econometric method.

### Online Reviews and Outpatient Visits

Numerous empirical studies suggest reputation is one of the predominant factors in influencing seller performance [36,37] and consistently reveals that there is a close relationship between reviews and future visits. Online reviews can improve the interaction between consumers and sellers and decrease consumer risk, thereby increasing trust and cooperation on both sides [38,39]. Positive reviews can also positively impact product demands [7,20]. Reviews are increasingly believed to influence consumer behavior [40] and be more effective than traditional advertising [41].

Online health care communities are changing how patients choose physicians. The digitization of health care reviews makes it easy for patients to find physician treatment information, assists them in thoroughly evaluating physicians before making a choice, increases their trust in the physician, and decreases perceived risk [42,43]. However, how reviews impact patient choice in health care has rarely been researched. In this paper, we investigated the role of online reviews in influencing patient decisions and thoroughly considered the endogeneity of online reviews. Previous studies have used different measurements about reviews, including the overall review rating [44-46] and the volume of online posting [47]. We hypothesized that both the overall review rating and number of reviews positively impact outpatient visits.

- H1a: An improvement in the overall review rating leads to an increase in outpatient visits on that website.
- H1b: An improvement in the number of reviews leads to an increase in outpatient visits on that website.

### Channel Effects and Moderating Effects

With the emergence of online communities, more and more physicians adopt multiple channels to serve patients. Existing studies from other fields find both complementary and substitute effects between online and offline channels. From the complementary perspective, a number of studies suggest that the internet has a distinct influence on offline sales [16,48]. Many product and service receivers still rely on offline stores for the actual product or service purchase. Because the internet gains increasing importance for information collection [48], online channels may have spillover effects, generating increased purchases in offline channels [16]. These studies emphasize the theoretical advantages of integrating online services with existing physical channels. For example, a combinations of channels can be used to target different kinds of service receivers and offer different kinds of services cost effectively [49]. From a substitution perspective, researchers suggest that there may be substitution by advertisers between print, television, and radio advertising channels [50,51].

By analyzing the existing studies, we believe that studies that find substitute effects often focus on these highly standardized products. Using the example of a cup, a seller can sell it online or in the store, and the buyer gets the same thing regardless of the channel chosen. Some product categories compete because they can serve a similar defining purpose and thus may have similar potential customers [52,53]. However, for the health care industry, diagnoses often cannot be given to patients using online services; only suggestions can be given. Online channels cannot provide services that are identical to offline channels. If patients choose to get advice online, they have to accept the risks associated with the fact that the doctor cannot communicate with them face-to-face or look directly at the patient, listen to verbal cues, examine the patient physically, or even use the four diagnostic methods of traditional Chinese medicine. We hypothesized, however, that there is a complementary effect between online services and outpatient visits in health care.

Online health care communities can help patients access information about and contact physicians [54]. Through written and telephone consultation services, patients can engage with physicians before going to hospitals. Online communication helps patients to get to know the physician, thus reducing their uncertainty and sense of risk and enhancing their trust in the physician and increasing outpatient visits. Based on these insights, we hypothesized that the more online services that patients use, the higher the use of outpatient visits.

- H2a: A physician who provides written consultation services has higher totals of outpatient visits.
- H2b: A physician who provides telephone consultation services has higher totals of outpatient visits.

A physician providing online services can give patients more opportunity to evaluate the, which can enhance patient trust. Online service content is public to all users of online health communities, so these public communications give patients some insight into the physician’s ability, including medical quality and bedside manner. Reviews are from patients who have finished an outpatient visit and can provide information to potential patients. If a physician provides online services, online service content offers a source of information for patients so they may be less dependent on reviews. If a patient communicates with a physician using online services before making an appointment for outpatient care, communication in advance can also decrease the uncertainty between physicians and patients.

During this channel extension process, consumer experiences with a seller in one channel may affect their perceptions and beliefs about the same seller in another channel [55]. The use of online services can reflect physician popularity and decrease the perceived risk of offline service, a similar effect of reviews, which are also described as a quality signal and can reduce perceived risk. Based on these considerations, we hypothesized that online services mitigate the relationship between reviews and outpatient visits.

- H3a: Increasing numbers of online written consultations by a physician mitigates the main effect between reviews and outpatient visits.
- H3b: Increasing numbers of online telephone consultations by a physician mitigates the main effect between reviews and outpatient visits.

## Methods

### Research Contexts

Our research contexts are Haodf and Guahao, two very popular and professionally regarded online health care communities in China that have established cooperative relationships with big companies such as Tencent, Sina, and Sohu.

**Figure 2:**
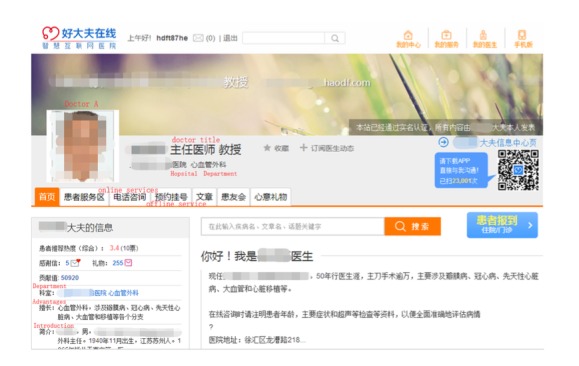
Haodf website.

Haodf was founded in 2006 and has become the most influential medical information and physician-patient interaction platform in China. On this platform, physicians can choose to offer online written consultations, telephone consultations, outpatient visits, or all of the above. Patients can search for generalized health information and/or ask physicians questions. Many unique attributes and services are available on Haodf to help patients make better and more accurate selections that suit their needs. Patients visit in increasing numbers and use this website to get help from physicians online. Haodf began to provide video consultation services in late 2016; however, only a very few patients use these services, so we did not consider them in our paper. Figure 2 shows a physician page on the Haodf website.

**Figure 3:**
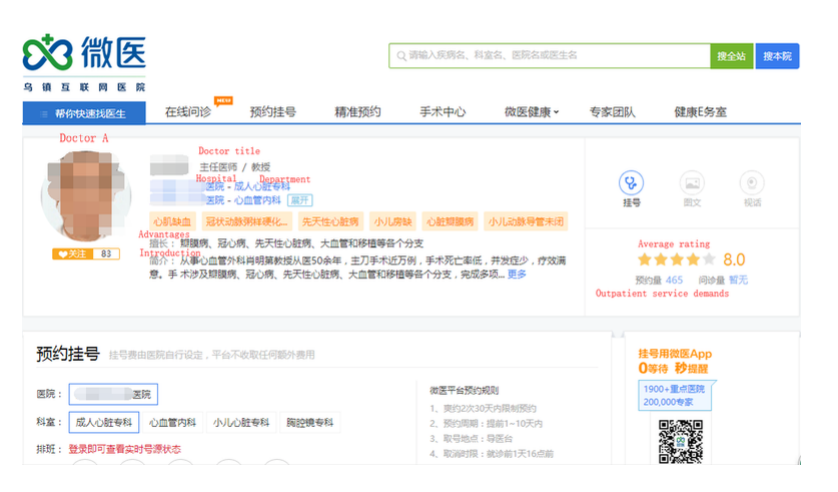
Guahao website.

Guahao was founded in 2010 and has become the leading online health care community for outpatient appointments specifically. Guahao was authorized by the China Health and Family Planning Committee in March 2010. With the help of Guahao, patients can make appointments easily, save valuable time, and increase efficiency. It has helped more than one hundred million people. Guahao began to provide online written consultations and video consultation services in September 2016. However, compared with outpatient appointments, the proportion of written and video consultation use is small. Our data were collected in 2014 when only outpatient care appointment service was provided on Guahao. Figure 3 shows a physician page on the Guahao website.

While Haodf is designed to help patients find suitable physicians to provide written and telephone consultations and outpatient visits, Guahao aims to provide people with the most efficient and best medical treatment and only provides outpatient appointments. These two communities automatically create homepages for physicians and their hospitals based on a directory collected. Physicians can choose to manage their homepages and work on them. Both websites have a formal and comprehensive reputation mechanism, which is important for this study. Patients can post their treatment experiences after receiving outpatient services, which helps potential patients make better choices.

### Sample and Data Collection

The homepage contains details of the physician, including their title (eg, chief physician, associate chief physician, attending physician), hospital that the physician belongs to, and the hospital’s level (eg, level A, B and C; level A offers the highest level of care). More importantly, it shows text content of all treatment experiences (reviews) and number of patients treated by online consultation, telephone consultation, and outpatient visit. The website also calculates the overall review rating for each physician based on all reviews.

We developed a crawler to automatically download homepages of physicians and information about physicians from Haodf and Guahao. For Guahao, we crawled the active physicians who have added or modified outpatient information or individual information, and for Haodf, we crawled physicians who are active and provided outpatient visits. We completed the collection process for three periods (one week each in June, September, and December 2014). We used a difference-in-difference method to compare physician outpatient visits on Haodf and Guahao, so we needed to determine which physicians had a homepage on both websites. Our physician samples needed to be present on both websites during all sampling periods; after we matched physicians from two websites and three time points, the number of observations shrank. A total of 474 physicians were included in our research using this criterion. For each physician in our sample, we gathered their corresponding service and review information at each time point.

We collected the following data from each physician’s homepage on both websites:

- All reviews for physician posted by patients until the day of our data collection, including the number of reviews for each physician and the overall review rating (on a scale of 0 to 10, 0 meaning very dissatisfied and 10 meaning very satisfied). The overall review rating reflects both medical quality and bedside manner of physician. Reviews on both websites come from patients who receives treatment at outpatient visits
- Number of outpatient visits for physician
- Number of online written and telephone consultations for physician (available on Haodf only)
- Date physician joined website (because length of time on the website can influence reviews, online services, and outpatient visits)

### Variables and Models

Our empirical variables are shown in Table 1. Dependent variables are the number of outpatient visits on both websites, which are easily obtained from the physician homepages and represent the performance of physicians. We took the logarithmic value of the number of reviews, online services, and outpatient visits to stabilize the variance. The number of outpatient visits on each website is a function of a physician fixed effect (*p_i_*), an online health care community website fixed effect (*w_i_*), and other factors like the number of reviews. A physician fixed effect is related to factors such as age, education, gender, medical title of the physician, level of the hospital that the physician belongs to, and popularity of the physician. The online health care community website fixed effect is related to website design and patient preference.

**Figure 4:**
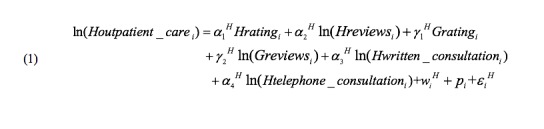
Equation for Haodf.

**Figure 5:**
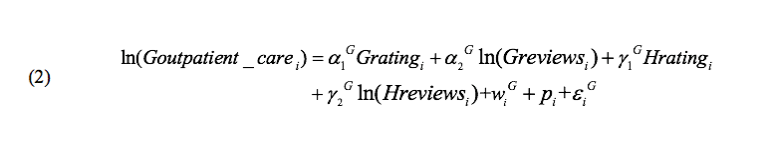
Equation for Guahao.

We used *Houtpatient_care* and *Goutpatient_care* to denote the number of outpatient visits, *Hreview* and *Greview* to denote the number of patient reviews on Haodf (Figure 4) and Guahao (Figure 5), respectively (we allow Haodf reviews to influence patients on Guahao and Guahao reviews to influence patients on Haodf). Similarly, *Hrating* and *Grating* respectively represent the summary statistics of a physician’s online reviews—the overall review rating. In addition, for Haodf, we consider two extra variables: *Hwritten_consultation* refers to the number of a physician’s online written consultations and *Htelephone_consultation* denotes the number of online telephone consultations. The superscripts *H* and *G* refer to Haodf and Guahao, respectively.

**Table 1:** Variable description.

Variable and symbol		Explanation
**Dependent variables**		
	*Ln(Houtpatient_care)*	Number of physician outpatient visits on Haodf (logarithmic form).
	*Ln(Goutpatient_care)*	Number of physician outpatient visits on Guahao (logarithmic form).
**Independent variables**		
	*Hrating*	Overall review rating of the physician on Haodf.
	*Grating*	Overall review rating of the physician on Guahao.
	*Ln(Hreview)*	Number of reviews on Haodf (logarithmic form).
	*Ln(Greview)*	Number of reviews on Guahao (logarithmic form).
	*Ln(Hwritten_Consultation)*	Number of physician online written consultations on Haodf (logarithmic form).
	*Ln(Htelephone_Consultation)*	Number of physician online telephone consultations on Haodf (logarithmic form).
**Moderating effects**		
	Hrating*Ln(Hwritten_Consultation)	Moderating effect of online written consultations on the relationship between reviews and outpatient visits.
	Grating*Ln(Hwritten_Consultation)	same
	Ln(Hreview)*Ln(Hwritten_Consultation)	same
	Ln(Greview)*Ln(Hwritten_Consultation)	same
	Hrating*Ln(Htelephone_Consultation)	Moderating effect of online telephone consultations on the relationship between reviews and outpatient visits.
	Grating*Ln(Htelephone_Consultation)	same
	Ln(Hreview)*Ln(Htelephone_Consultation)	same
	Ln(Greview)*Ln(Htelephone_Consultation)	same
**Control variables**		
	*Htime*	Opening date of physician homepage on Haodf.
	*Gtime*	Opening date of physician homepage on Guahao.

**Figure 6:**
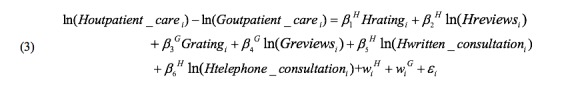
Equation to eliminate physician fixed effects.

We expect there are unobservable factors (fixed effects) that may affect the independent or dependent variable and cause a deviation of estimation if omitted. The physicians we collected are matched; for example, consider physician *i* in our dataset—although they work on both websites and have homepages on both websites, they are exactly the same person and have exactly the same characteristics such as title, popularity, etc (*p_i_^H^*=*p_i_^G^*). We are able to control for the possible effect of unobserved physician characteristics on both reviews and outpatient visits and can eliminate physician fixed effects by differencing the data across websites (Figure 6).

**Figure 7:**
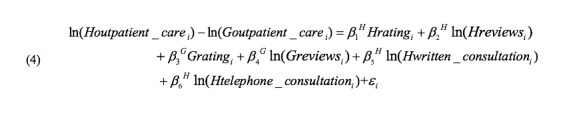
Equation to eliminate online health care community fixed effects.

For the online health care communities fixed effect, we first assume that both websites are virtually identical in terms of patient preference (ie, *w_i_^H^*=*w_i_^G^*), so we eliminate website fixed effects by differencing the data across websites (Figure 7).

**Figure 8:**
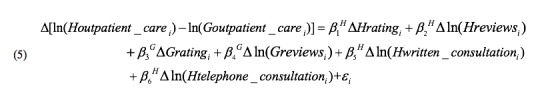
Equation to eliminate differences across the websites.

However, if there are differences across the two websites (ie, μ*_i_^H^*≠μ*_i_^G^*), we need to collect data for another time point and difference the data across the websites and time (Figure 8).

All the above equations omit interaction terms. Accordingly, we add the moderating effects (Figures 7 and 8) in our empirical models. Formula expression is omitted to save space.

## Results

### Descriptive Statistics and Correlations

Tables 2 and 3 show the summary, description, and correlation of our variables. From Table 2, we can see there are obvious changes for all variables, which is helpful for empirical analysis. There are a few notable differences across the two websites that are apparent in Table 2. First, the mean of the difference between the number of outpatient visits on Haodf and Guahao is less than zero. This is consistent with the primary functions of the websites: Haodf provides many services for patients to choose, and its primary services are online services; Guahao specializes in providing outpatient care appointments. Second, Haodf has more reviews than Guahao. Third, the overall review rating is higher at Haodf; although again, they are overwhelmingly positive overall at both websites.

From Table 3, we can see the number of online written and telephone consultations positively impacts the difference between the number of outpatient visits of physicians on Haodf and Guahao. We can also see that the number of reviews on Haodf is positively related to the difference in the number of outpatient visits on the two websites. However, the overall review rating and number of reviews on Guahao are negatively related to the difference in outpatient visits on the two websites.

**Table 2:** Summary data.

Variable	Jun 2014 mean (standard error)		Sep 2014 mean (standard error)		June-December 2014 mean (standard error)	
	Haodf	Guahao	Haodf	Guahao	Haodf	Guahao
*Lnrating*	9.084 (2.560)	8.084 (2.072)	9.284 (2.220)	8.353 (2.141)	—	—
*Lnreviews*	3.450 (1.070)	2.893 (1.661)	3.483 (1.041)	3.103 (1.616)	—	—
*Lnwritten consultation*	6.363 (1.749)	—	6.485 (1.663)	—	—	—
*Lntelephone consultation*	1.781 (1.931)	—	1.987 (1.998)	—	—	—
*LnHoutpatient_care-LnGoutpatiet_care*	–1.380 (2.370)	—	—	—	–1.662 (2.209)	—

**Table 3:** Description and correlation.

Variable	1	*P* value	2	*P* value	3	*P* value	4	*P* value	5	*P* value	6	*P* value
*1. LnHoutpatient_care-LnGoutpatiet_care*	—	—	—	—	—	—	—	—	—	—	—	—
*2. LnHwritten_consultation*	0.323	.04	—	—	—	—	—	—	—	—	—	—
*3. LnHtelephone_consultation*	0.195	.03	0.453	.02	—	—	—	—	—	—	—	—
*4. Hrating*	0.037	.22	0.184	.01	0.051	.34	—	—	—	—	—	—
*5. LnHreview*	0.225	.03	0.577	.01	0.419	.02	0.278	.01	—	—	—	—
*6. Grating*	–0.316	.02	–0.060	.23	0.065	.21	–0.034	.32	0.034	.43	—	—
*7. LnGreview*	–0.660	.03	0.110	.07	0.102	.07	0.070	.44	0.222	.02	0.359	.03

### Empirical Results

#### Results Without Considering the Website-Specific Fixed Effects

We used an ordinary least squares regression model for analysis using STATA (StataCorp LLC) software. We first assume there were no website-specific fixed effects and examined the model (Figure 7). Table 4 shows the estimation results. Column 1 presents the results of the control variables. As we chose physician *i*, who provides services on both websites, the physician individual characteristics did not need to be considered. We included the opening time (duration of use) for each physician in column 1. A longer time on the website may lead to having more patients and affect important variables in our model. Columns 2 and 3 introduce these independent variables. Both written and telephone consultations increase the difference in outpatient visits (written consultation: β=0.325, *P*<.001; telephone consultation: β=0.093, *P*=.005), and this suggests there are complementary effects between online services and outpatient visits. Physicians can use online services to attract more patients to have treatment in hospitals. The coefficient of the number of reviews on Haodf is positive and statistically significant (β=0.518, *P*<.001), suggesting that when reviews increase, visits on Haodf becomes larger. However, the ratings on Haodf do not significantly impact the difference in outpatient visits. The overall review rating on Haodf is 9.084, which is very high compared with the full mark (ie, 10). High overall review ratings make it more likely patients will discount the reviews and not use them for decision making. Again, when ratings and number of reviews rise on Guahao, the difference in outpatient visits decreases (ie, outpatient visit increases on Guahao relative to Haodf; rating: β=–0.066, *P*=.009; number of reviews: β=–1.037, *P*<.001). The absolute value of the coefficient of the number of reviews on Guahao is bigger than on Haodf, suggesting that difference in visits responds more to the number of reviews on Guahao than on Haodf. This is consistent with the main function of the two sites. Guahao only provides outpatient care appointments, and patients can only refer to the reviews from other patients to make choices. However, in addition to offline services, Haodf also provides online services, so there is more information for patients to make choices.

Column 4 in Table 4 includes the interaction effects. As the impact of ratings on Haodf is not significant, we only introduce the interaction terms of significant factors. Online services negatively moderate the relationship between the number of reviews on Haodf and difference in visits (written consultations and reviews: β=–0.101, *P*<.001; telephone consultations and reviews β=–0.011, *P*=.04). However, the moderating effects are not statistically significant for the number of reviews on Guahao. When a physician provides online services, the impact of ratings on Guahao on difference in visits declines (written consultations: β=0.019, *P*=.01; telephone consultation: β=0.033, *P*=.04). The adjusted *R*^2^ is 64.2%; these variables explain the independent variable well.

**Table 4:** The effect of online services and reviews on outpatient visits (sample is the complete June 2014 sample. Dependent variable is the difference between the log outpatient visits on Haodf and the log outpatient visits on Guahao. Dependent variable is Ln(Houtpatient_carei)-Ln(Goutpatiet_carei).

Variable	Model 1 Coefficient (robust standard error)	*P* value	Model 2 Coefficient (robust standard error)	*P* value	Model 3 Coefficient (robust standard error)	*P* value	Model 4 Coefficient (robust standard error)	*P* value
*HTime*	0.289 (0.058)	.11	0.424 (0.055)	.11	0.036 (0.035)	.11	0.108 (0.065)	.04
*GTime*	–0.154 (0.107)	.12	–0.086 (0.102)	.12	0.102 (0.066)	.12	0.035 (0.035)	.12
*LnHwritten_Consultation*	—	—	0.397 (0.066)	<.001	0.325 (0.048)	<.001	–0.191 (0.197)	.11
*LnHtelephone_Consultation*	—	—	0.075 (0.060)	.10	0.093 (0.039)	.005	0.397 (0.219)	.04
*Hrating*	—	—	—	—	–0.028 (0.027)	.23	–0.003 (0.028)	.22
*LnHreview*	—	—	—	—	0.518 (0.081)	<.001	0.116 (0.244)	.03
*Grating*	—	—	—	—	–0.066 (0.034)	.009	–0.163 (0.143)	.04
*LnGreview*	—	—	—	—	–1.037 (0.043)	<.001	–1.119 (0.170)	<.001
LnHwritten_Consultation*LnHreview	—	—	—	—	—	—	–0.101 (0.039)	<.001
LnHtelephone_Consulation*LnHreview	—	—	—	—	—	—	–0.011 (0.039)	.04
LnHwritten_Consultation*Grating	—	—	—	—	—	—	0.019 (0.021)	.01
LnHwritten_Consultation*LnGreview	—	—	—	—	—	—	0.015 (0.028)	.45
LnHtelephone_Consulation*Grating	—	—	—	—	—	—	0.033 (0.206)	.04
Lntelephone_Consulation*LnGreview	—	—	—	—	—	—	–0.001 (0.026)	.31
Adjusted *R*^2^	0.0002	—	0.101	—	0.634	—	0.642	—
N	474	—	474	—	474	—	474	—

#### Results With Considering the Website-Specific Fixed Effects

The websites have different characteristics, so omitting the website-specific fixed effects may bias the estimation results. In this section, we estimate the equation seen in Figure 8. The results are shown in Table 5.

The homepages for all 474 physicians on both websites existed during the second period. Columns 1 and 2 on Table 5 include the independent variables. The coefficients of the number of reviews are higher in magnitude than on Table 4, even though some are no longer significant. The impacts of ratings on both websites are not significant. This may be due to relatively little variance in the overall review rating over time. Most of the results of the previous section are replicated. Thus, there are complementary effects between online services and visits (written consultations: β=0.172, *P*=.03; telephone consultations: β=0.155, *P*<.001), and therefore hypotheses H2a and H2b are supported. An increase in the number of reviews on Haodf over time results in a higher number of visits to the physician on Haodf over time (β=0.588, *P*<.001); the same is true for the number of reviews on Guahao (β=–1.661, *P*<.001), supporting hypothesis H1b.

Column 3 on Table 5 shows the results of moderating effects. We only introduce the moderating effects of significant factors. The results are almost the same as we predicted (hypothesis H3b is partly supported), except the moderating effect between telephone consultations and number of reviews on Haodf is not significant. When a physician provides online written consultations, the impact of reviews declines (β=–0.829, *P*=.004). When a physician provides online services, the impact of reviews on Guahao for visits declines (written consultations: β=0.730, *P*=.03; telephone consultations: β=0.296, *P*=.009). The adjusted *R*^2^ is 42.7%, which has declined compared with the same value on Table 4.

**Table 5:** The effect of changes in online services and reviews on changes in visits over 2 months (sample is the set of physicians who were available on both websites in June and December 2014. Reviews were collected in June and September 2014. Dependent variable is Δ[Ln(Houtpatient_carei)-Ln(Goutpatiet_carei)]).

Variable	Model 1 Coefficient (robust standard error)	*P* value	Model 2 Coefficient (robust standard error)	*P* value	Model 3 Coefficient (robust standard error)	*P* value
*ΔHrating*	–0.001 (0.021)	.50	–0.001 (0.208)	.50	–0.003 (0.208)	.50
*ΔLnHreview*	0.591 (0.127)	<.001	0.588 (0.126)	<.001	0.655 (0.143)	<.001
*ΔGrating*	0.049 (0.049)	.12	0.043 (0.048)	.13	0.049 (0.048)	.13
*ΔLnGreview*	–1.643 (0.097)	—	–1.661 (0.096)	<.001	–1.827 (0.119)	<.001
*ΔLnHwritten_Consultation*	—	—	0.172 (0.097)	.03	0.175 (0.151)	.04
*ΔLnHtelephone_Consultation*	—	—	0.155 (0.058)	<.001	0.049 (0.073)	.03
ΔLnHwritten_Consultation*ΔLnHreview	—	—	—	—	–0.829 (0.359)	.004
ΔLnHtelephone_Consultation*ΔLnHreview	—	—	—	—	–0.057 (0.349)	.54
ΔLnHwritten_Consultation*ΔLnGreview	—	—	—	—	0.730 (0.567)	.03
ΔLnHtelephone_Consultation*ΔLnGreview	—	—	—	—	0.296 (0.156)	.009
Adjusted *R*^2^	0.39	—	0.411	—	0.427	—
N	474	—	474	—	474	—

### Robustness Check

We examine the robustness of our estimations in Tables 4 and 5. For Table 4, we repeat the specification of column 4, but we examine only the subsample of 400 physicians who have at least one review on each website. The results are shown in column 1 on Table 5 and are similar to those we presented previously. All signs of the coefficients are as we predicted. For Table 5, we only include physicians who have at least one review variable changed; we repeated the equation found in Figure 8 by using the subsample 371, and the results are shown in column 2 on Table 6. The results prove the robustness of our empirical results.

**Table 6:** Robustness check results (for column 1, sample is the subsample of physicians who had at least one review on both websites in June 2014, and dependent variable is Ln(Houtpatient_carei)-Ln(Goutpatiet_carei). For column 2, sample is the subsample of physicians who had new reviews posted on both websites between June and September 2014).

Variable	Model 1 Coefficient (robust standard error)	*P* value	Model 2 Coefficient (robust standard error)	*P* value
*HTime*	0.104 (0.064)	.01	—	—
*GTime*	0.035 (0.034)	.22	—	—
*LnHwritten_Consultation*	–0.035 (0.205)	.46	0.189 (0.020)	.01
*LnHtelephone_Consultation*	0.237 (0.204)	.03	0.067 (0.012)	.03
*Hrating*	0.008 (0.033)	.43	0.001 (0.002)	.43
*LnHreview*	0.219 (0.278)	.04	0.738 (0.201)	<.001
*Grating*	–0.023 (0.143)	.04	0.002 (0.024)	.06
*LnGreview*	–1.083 (0.143)	<.001	–0.205 (0.121)	<.001
LnHwritten_Consultation*LnHreview	–0.105 (0.044)	<.001	–0.988 (0.273)	.009
LnHtelephone_Consulation*LnHreview	–0.006 (0.038)	.03	–0.105 (0.023)	.05
LnHwritten_Consultation*Grating	0.004 (0.022)	.04	—	—
LnHwritten_Consultation*LnGreview	0.027 (0.032)	.32	0.870 (0.556)	.03
LnHtelephone_Consulation*Grating	0.012 (0.019)	.03	—	—
Lntelephone_Consulation*LnGreview	–0.015 (0.027)	.32	0.443 (0.154)	.03
Adjusted *R*^2^	0.613	—	0.408	—
N	400	—	371	—

## Discussion

### Principal Findings

We studied the role of reviews in the health care industry and found that the number of reviews tended to have positive impacts on both websites. Our empirical results show that patients value the number of reviews more than the average rating when making decisions. The evidence suggests that physicians should try to improve their service quality and attitude to attract more patients to write reviews for them. Our regression estimates show that the relative visits of a physician across the two websites are related to the differences across the websites in the number of reviews.

When we used the equation found in Figure 8 to eliminate the physician- and website-specific fixed effects, the effects of overall review ratings for both websites were no longer significant. This finding differs from prior studies, which generally saw significant and positive effects of the overall review rating [44-46]. First, we found the overall review rating was very high on both websites—much higher than in other fields such as e-commerce. One possible explanation is that the health care industry in China is facing intense physician-patient conflicts [56]. Possible manipulations in reviews on websites may exist [57] such as deleting negative reviews. Another possible explanation is that many diseases (eg, chronic diseases) require follow-up, and patients may be afraid of being retaliated against by physicians they review poorly. From the data summary on Table 3, we show that the mean value of the overall review rating is over 9.0 on Haodf and 8.0 on Guahao, which may seem artificially inflated to patients. Second, we use a more sophisticated econometric method, difference-in-difference, to eliminate physician- and website-specific fixed effects, which may not have been fully addressed in other studies.

For channel effects, our results show that online services complement offline services (outpatient visits), which is consistent with our hypotheses. Online services can help patients get more information but cannot replace face-to-face service. By first having a written or telephone consultation with a physician, patients gain a basic understanding of their disease, and then they can see the physician in the hospital for further details.

**Figure 9:**
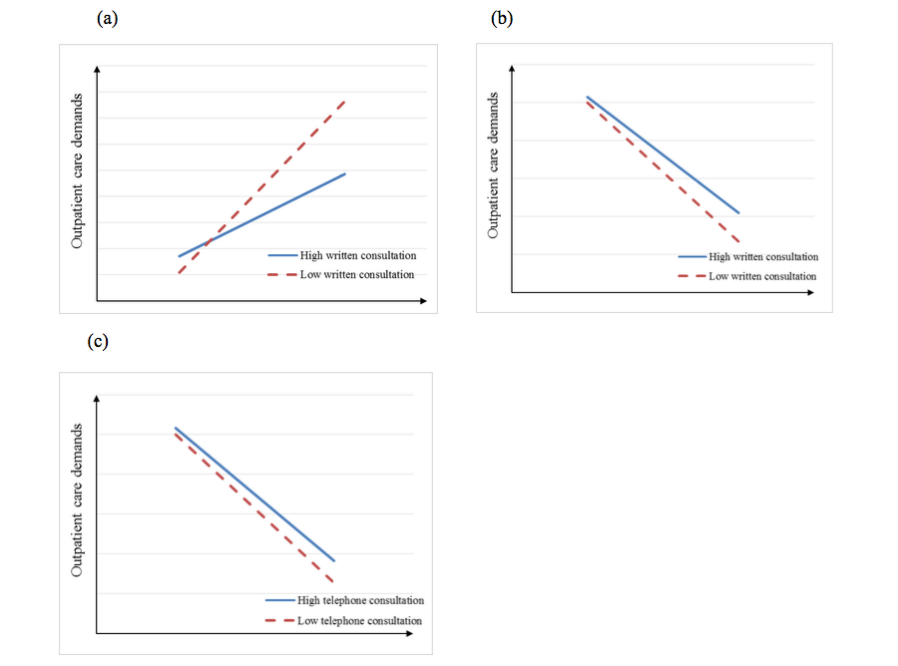
Images (a) and (b) show the moderating effects of written consultations on the relationship between the number of reviews on Haodf/Guahao and outpatient visits. Image (c) shows the moderating effect of telephone consultations on the relationship between the number of reviews on Guahao and outpatient visits.

For the interaction effects, our results show that online services mitigate the relationship between reviews and outpatient visits (Table 5). The interaction effects are illustrated in Figure 9. We only draw the moderating effects in Table 5. Haodf provides online written and telephone consultations. We show that these two kinds of services significantly affect outpatient visits in our empirical results, and this eliminates the impact of reviews of Haodf on patient choice.

### Limitations

This paper has several limitations. First, we do not explore the context of the reviews. We just study the overall review rating and number of reviews, and this could affect the impact of reviews. For example, some reviews contain more information, and these kinds of reviews may have more impact. Second, we studied only two contexts. This helped us improve the internal validity, but it may have also reduced the generalizability of our findings, and future research should validate our results in more service contexts. Future research can take more effective empirical methods to solve these limitations.

### Implications of the Research

Our study has important theoretical and practical implications. For the theoretical implications, our study enriches the research on the role of reviews by investigating them in the health care field. Existing studies mainly research them in the marketplace. Moreover, we have addressed the endogeneity of reviews—self-selected problem and the impact of underlying factors (eg, service quality) in our paper. We use a difference-in-difference method to account for physician- and website-specific effects. Our paper has important theoretical implications for research in health care. Second, despite some studies indicating that there are channel effects in the marketplace [12,16], literature rarely uses empirical methods to validate claims. Our study is among the first to use real data to empirically examine the channel effect, especially in health care, which is a universally beneficial sector. The research contexts allow us to study the effects of two kinds of online channels on offline channels, and our results show that there are channel effects in health care. Third, our study contributes to existing theories of reviews and channel effects by hypothesizing and empirically testing the moderating influence of online channels on the relationship between online reviews and offline channels. In analyzing existing literature, we found that there were few studies combining them. Although some researchers have studied the importance of reviews [7,20], few studies consider their effect on the relationship between online and offline channels.

This paper also makes contributions to practice. First, multichannel use is on the rise, with practitioners seeking guidance on how to balance different channels. Our empirical results show that a multichannel strategy is helpful for physicians to access more patients. Therefore, we believe our analysis provides insights that are helpful to physicians as they consider implementing a multichannel strategy such as providing online consultation services to patients. Second, based on the empirical results, our study gives physicians suggestions to improve their reviews (both medical quality and bedside manner), such as learning more to improve medical skills. Moreover, we found that the overall review rating is not always effective in influencing patient decisions and recommend that physicians encourage and remind patients to write reviews for them. Third, our study highlights the importance of rethinking the nature of reviews in relation to multichannel strategies. Our study shows online services have a significant moderating effect on the relationship between reviews and outpatient visits. This result indicates that even if physicians have lower reviews, they can improve their career outcomes by working hard in an online health care community. Our study has proved that online health communities benefit not only patients but also physicians. These results can encourage physicians to attract more patients and achieve their career goals by participating in online communities.

### Conclusions

A majority of empirical work has examined the role of online reviews in consumer decisions. However, less evidence has been found in health care, and endogeneity of online reviews has not been fully considered. Moreover, the important factor of physician online services has been neglected in patient decisions. To address this research gap, this study investigates the effect of reviews and channel effects in health care by conducting a difference-in-difference analysis on two online health care communities. Our empirical results show that compared with average rating, patients consider number of reviews more when making decisions. The evidence suggests that physicians should try to improve their service quality and attitude to attract more patients to write reviews for them. Our regression estimates show that the number of visits to a physician across the two websites is related to the differences across the websites in the number of reviews. Our findings provide basic research on online health care communities and have both theoretical and practical implications.
